# Skilled Nursing Facility Performance and Readmission Rates Under Value-Based Purchasing

**DOI:** 10.1001/jamanetworkopen.2022.0721

**Published:** 2022-02-28

**Authors:** Robert E. Burke, Yao Xu, Liam Rose

**Affiliations:** 1 Division of General Internal Medicine, Perelman School of Medicine, University of Pennsylvania, Philadelphia; 2 Center for Health Equity Research and Promotion, Corporal Michael J. Crescenz Veterans Affairs Medical Center, Philadelphia, Pennsylvania; 3 Leonard Davis Institute of Health Economics, University of Pennsylvania, Philadelphia; 4 Health Economics Resource Center, Department of Veterans Affairs, Palo Alto, California; 5 Stanford-Surgery Policy Improvement Research & Education Center, Stanford University, Stanford, California

## Abstract

**Importance:**

The new Medicare Skilled Nursing Facility Value-Based Purchasing program (SNF VBP) seeks to improve patient outcomes by awarding financial incentives or penalties based on 30-day hospital readmission rates. Skilled nursing facilities (SNFs) can avoid a penalty through low baseline readmission rates or improvement over time.

**Objective:**

To assess the baseline performance and improvement over time of SNFs in the SNF VBP program.

**Design, Setting, and Participants:**

This cross-sectional study examined readmission rates, financial penalties and incentives, and facility and patient characteristics associated with these outcomes at 14 959 US SNFs that received Medicare payments between January 1, 2015, and December 31, 2019.

**Main Outcomes and Measures:**

Outcomes were readmission rates and financial penalties by facility. The SNFs were classified as improvers in the analysis if they had better improvement scores than baseline scores under the program and achievers if they had higher baseline scores than improvement scores.

**Results:**

Of 14 959 SNFs studied, 1849 (12.3%) were assigned their improvement score as their performance score in the first year of the program. Of these, 1167 (63.1%) received a financial penalty, whereas 374 (20.2%) received a bonus. Only 52 facilities that performed poorly at baseline (0.3% of all SNFs and 0.7% of below-median performers) were able to improve enough to avoid a financial penalty, despite large improvements in readmission rates. Improver SNFs treated larger racial minority populations (mean [SD], 74.57% [23.42%] White in the improver group vs 79.15% [22.18%] in the achiever group) and were located in counties with larger minority populations (mean [SD], 15.48% [14.05%] Black in the improver group vs 11.57% [12.72%] Black in the achiever group). The most important predictors of improvement were related to SNF finances, such as operating margin (mean [SD], −0.74 [13.87]) and occupancy rates (mean [SD], 79.93 [14.81]).

**Conclusions and Relevance:**

This cross-sectional study suggests that the SNF VBP program did not offer a viable path for nearly all low-performing SNFs to avoid financial penalties through improved readmission rates.

## Key Points

**Question:**

In the Medicare Skilled Nursing Facility Value-Based Purchasing program, were low-performing skilled nursing facilities at baseline able to improve readmissions enough to avoid financial penalties?

**Findings:**

In this cross-sectional study of 14 959 skilled nursing facilities, only 0.7% of facilities that performed poorly at baseline were able to improve readmission rates enough to avoid a financial penalty.

**Meaning:**

The Medicare Skilled Nursing Facility Value-Based Purchasing program may not be offering sufficient incentives for improvement for low-performing facilities, particularly those in areas with traditionally underserved populations.

## Teaser

This cross-sectional study assesses changes in readmission rates at skilled nursing facilities under Medicare’s value-based purchasing program.

## Introduction

One in 5 Medicare beneficiaries discharged from the hospital receives postacute care in a skilled nursing facility (SNF) at a cost of more than $28 billion annually.^1^ Nearly one-quarter of those admitted to SNFs are readmitted to the hospital within 30 days,^2^ and readmission is associated with a quadrupled mortality rate within 6 months.^3^ The Skilled Nursing Facility Value-Based Purchasing (SNF VBP) program is the first national value-based purchasing program for postacute care. This program aims to reduce readmission rates by withholding 2% of all Medicare fee-for-service SNF revenues and redistributing a portion as incentive payments tied directly to an SNF’s all-cause, unplanned, 30-day hospital readmission rate. Financial incentives can range from SNFs losing the full 2% penalty (all the funds held back are not returned) to gaining a 2% bonus (on top of receiving their 2% withhold back) via an incentive multiplier, calculated based on a performance score.

An important part of the SNF VBP program design is that it allows SNFs 2 different opportunities to earn a high performance score and avoid financial penalties. First, SNFs can be sufficiently high-performing relative to their peers in the baseline year (known as the achievement score). The achievement score is an across-SNF comparison. Second, SNFs can show relative improvement between the baseline year and performance year (2 years later), known as the improvement score. The improvement score is a within-SNF comparison. Whichever score is higher (achievement vs improvement) is assigned as the performance score, which determines how much financial penalty or bonus SNFs receive. Thus, all SNFs can be thought of as improvers or achievers based on which score they use. The program began with a baseline year in 2015 followed by a performance year in 2017, and financial penalties and incentives were first applied in 2019.

The intent of the improvement pathway is to provide an incentive for SNFs far from the achievement threshold to improve, rather than only rewarding consistently high-performing facilities. Even SNFs that have historically underperformed in terms of readmission rates could earn nearly the full bonus, starting a virtuous cycle of improvement and financial reward. Rewarding improvement could therefore also enhance health equity, given substantial evidence that historically underperforming SNFs disproportionately serve racial and ethnic minority populations in poorer areas.^4,5,6^ Early evaluations suggested that three-quarters of SNFs in the US received a financial penalty in 2019, which increased in 2020.^7,8^ Furthermore, penalized SNFs were more likely to serve vulnerable populations.^9^

However, prior studies have not disentangled the differences between achievers—defined as those that had higher baseline scores than improvement scores—and improvers—defined as those with higher improvement scores than baseline scores. Because the intent of the SNF VBP program is to improve SNF readmission rates and not simply reward high performers, describing SNFs that were assigned their improvement scores as their performance scores—particularly those with the largest improvements—may offer insights on how to better design programs that drive improvements in outcomes. We thus sought to describe the improvers in the SNF VBP program in terms of facility-, patient-, and area-level characteristics.

## Methods

### Sample Selection

For this cross-sectional study, we used the same inclusion and exclusion criteria as the SNF VBP program and focused on the first year that financial incentives and penalties were assessed (fiscal year 2019). All Medicare-certified SNFs (ie, 97% of all SNFs in the US) participate in the SNF VBP program, including swing bed facilities. The Centers for Medicare & Medicaid Services (CMS) award incentives are based on Part A fee-for-service payments. The all-cause, all-condition adjusted readmission measure used in the SNF VBP program is broad and extends 30 days from hospital discharge regardless of whether the patient has been discharged from the SNF. The measure excludes Medicare Advantage beneficiaries, any observation stays in the hospital, SNF stays that begin more than 1 day after hospital discharge, and planned hospital readmissions. The SNFs with very low sample sizes are automatically returned the full amount withheld. More information about the SNF VBP program is included in the eAppendix in the Supplement, and the full specification of the readmission measure is publicly posted on the CMS website.^10^ The study was approved by the University of Pennsylvania Institutional Review Board. Informed consent and data deidentification were not required because the study did not include patient-level data. The study followed the Strengthening the Reporting of Observational Studies in Epidemiology (STROBE) reporting guideline.

### Data Sources

A full list of data sources, descriptions, and variables used is included in eTable 1 in the Supplement. Briefly, the SNF VBP data (including risk-standardized readmission rates in the baseline and achievement periods, improvement scores, achievement scores, performance scores, and incentive multipliers) were obtained from the CMS website. Data regarding SNF characteristics were compiled from the CMS Provider of Services files, LTCfocus,^11^ and publicly available CMS data regarding Nursing Home Compare star ratings and SNF cost reports in 2018. County-level characteristics were derived from the 2015 US Census Residential Population Estimates.^12^ Medicare Advantage penetration was calculated using publicly available enrollment data reported on the CMS website.^13^ We used the Post-Acute Care and Hospice Provider Utilization and Payment Public Use Files to estimate the size of the penalties in dollar terms.

### Descriptors

We selected characteristics of interest based on prior literature^7,8,9^ regarding associations with SNF readmission rates. These characteristics included (1) SNF ownership characteristics (whether the SNF was multifacility, hospital based, for profit, not for profit, or owned by the government, and the number of ownership changes in the past year); (2) financial indicators, including net operating margin (calculated as a ratio of the net operating profit or loss [numerator] to the operating revenue [denominator]), occupancy rate, and proportion of Medicaid bed-days; (3) treated patient population (age, race and ethnicity, number of deficiencies in activities of daily living, and case-mix index); (4) treatment characteristics (SNF length of stay and number of admissions per bed as a marker of turnover); and (5) performance on other quality metrics (Nursing Home Compare overall star ratings and individual components, including staffing, quality, and inspection star ratings). We found data regarding facility-level non-White race and ethnicity data to have significant missingness in LTCFocus; therefore, we report the proportion of White patients (which had much fewer missing data) and assume the remainder are patients of other races and ethnicities. Race and ethnicity data are important to report because nursing homes are known to exhibit disparities in access and quality by race, and an important question is to what extent the SNF VBP program affected these facilities. We also calculated characteristics of the county where the SNF is located, including urban or rural status, Herfindahl-Hirschman Index (calculated using the total number of SNF beds at the county level), county-level Medicare Advantage penetration, county-level demographic characteristics, and Area Deprivation Index,^14^ aggregated from zip code to county level and calculated as a mean of zip code–level scores.

### Statistical Analysis

We first compared SNFs that were assigned their improvement score (improvers) with those that were assigned their achievement score (achievers) as their performance score (ie, had higher improvement than achievement scores). Those with equal improvement and achievement scores were considered achievers. We calculated standardized mean differences to derive more meaningful comparisons given the large sample size. For ease of interpretation, we divided standardized differences into 3 groups, with standardized differences of greater than 0.2 (absolute difference) considered large, differences between 0.10 and 0.19 considered moderate, and differences less than 0.10 considered minimal differences between groups. These differences were displayed using an ordered forest plot for ease of interpretation.

We then plotted the distribution of achievement and improvement scores of all SNFs in our sample stratified by quartile of achievement score (in other words, by their baseline readmission rates). Although award incentives were a continuous multiplier, we marked SNFs by their use of improvement or achievement score and by their financial penalty or bonus achieved in 2019 (incentive multipliers of <1 were labeled as penalty and >1 as bonus). This approach was intended to identify the distribution of improvers across different baseline achievement levels and to describe the distribution of SNFs using their improvement score and earning a financial bonus doing so. We note that by design, improvement scores were capped at 90 rather than 100 to avoid a higher award payment for a low-performing SNF that improved greatly than a better-performing SNF that did not improve. We also compared characteristics of SNFs using their achievement score vs those using their improvement score within each quartile of achievement score.

On the basis of these results, we then described SNFs in the top quartile of improvement scores (the top improvers, which exhibited the most improvement in the sample). We also described the subgroup of top improvers that were originally low performers: those that had achievement scores below the median in their baseline year. To examine how each group varied from sample means, we compared the characteristics of both groups with those of the full sample. To contextualize the impact of the penalties, we estimated 2% of the total Medicare payments for fiscal year 2017 for the lowest and highest quartiles of SNFs by achievement score.

Finally, to determine predictors of improvement under the SNF VBP program, we used a common machine learning algorithm—generalized random forest plots. We used this approach because it effectively segregates characteristics based on their relative importance while still making it possible to explore interaction terms and minimizing concerns about collinearity. Conceptually, this algorithm divides the sample into progressively smaller groups that maximize homogeneity within groups and heterogeneity across groups, using observed covariates and interaction terms.^15^ We display the characteristics with the highest predictive power for an SNF being an improver (vs an achiever) using a variable importance plot. This plot demonstrates the relative importance of the variables of interest to one another in predicting whether a SNF is an improver (vs an achiever). All analyses were performed in Stata, version 16.1 (StataCorp LLC) and R, version 3.6.3 (R Foundation for Statistical Computing).

## Results

Of 14 959 SNFs in our sample, 1849 (12.3%) were assigned their improvement score as their performance score in the first year of the program. Of these, 1167 (63.1%) received a financial penalty, whereas 374 (20.2%) received a bonus. At baseline (2015), improvers had higher 30-day readmission rates than achievers (22% vs 19%; standardized mean difference, −2.5; 95% CI, −2.55 to −2.44) but this difference was attenuated in the performance year (2017) (20.0% vs 19.0%; standardized mean difference, −0.19; 95% CI, −0.24 to 0.14). Improvers had higher performance scores than achievers (37.8 vs 33.6; standardized mean difference, −0.15; 95% CI, −0.20 to −0.10), but this did not result in a large difference in mean incentive multiplier under the program (0.99, reflecting a 1% penalty; standardized mean [SD] difference, −0.09; 95% CI, −0.13 to −0.04). We estimated that SNFs in the lowest quartile of achievement scores would receive a penalty of approximately $36 000, whereas an SNF in the highest quartile of achievement scores would receive of bonus of $38 000.

**Figure 1.**
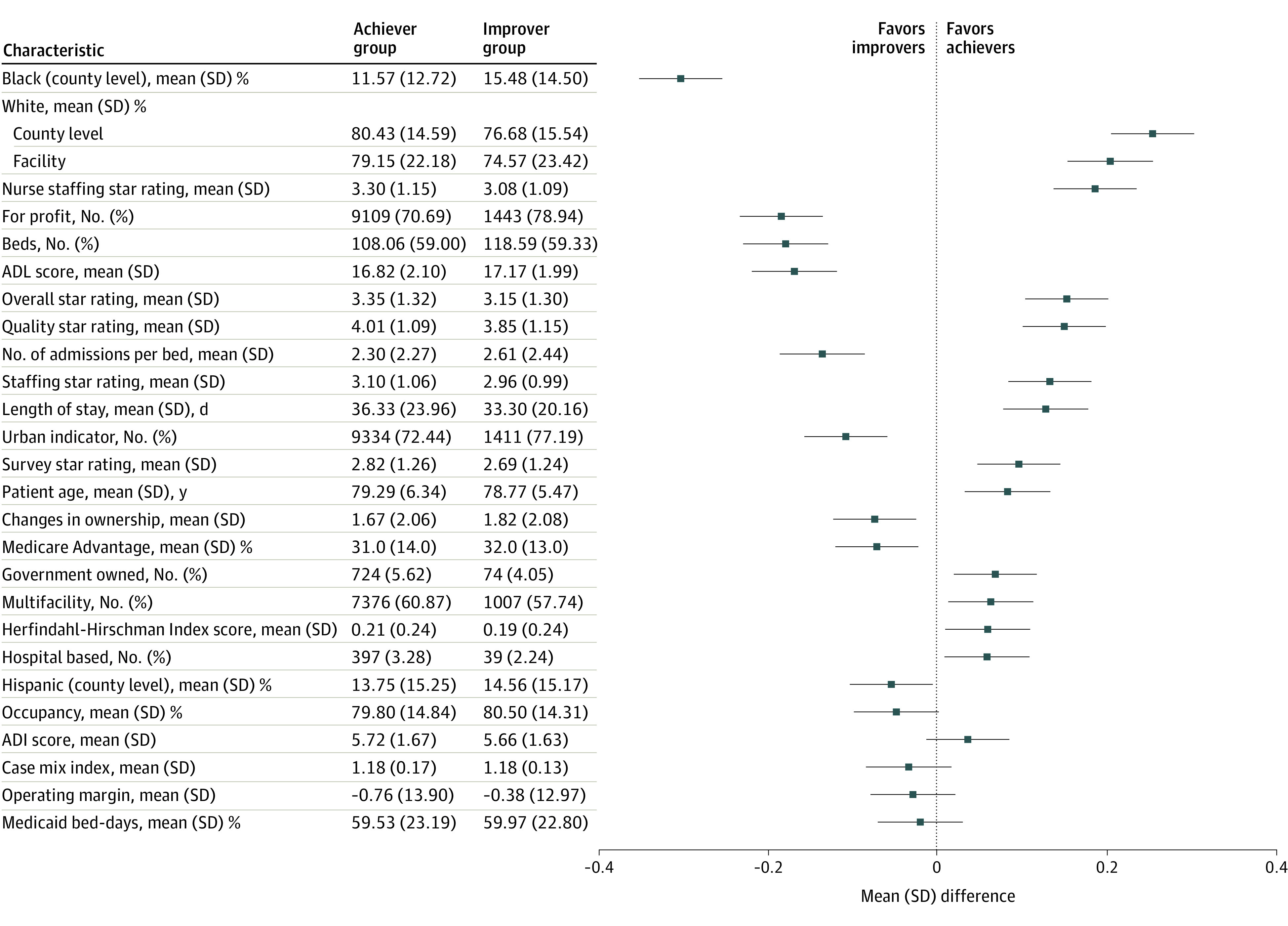
Characteristics of Improvers and Achievers in the Skilled Nursing Facility (SNF) Value-Based Purchasing Program Means (SDs) and numbers (percentages) of key SNF characteristics for the achiever and improver groups. The plot on the right side represents the standardized mean difference between the 2 groups for each characteristic. Error bars indicate SDs. ADI indicates Area Deprivation Index; ADL, activities of daily living.

The largest differences in improvers compared with achievers were in the racial makeup of the counties where SNFs were located (mean [SD] 15.48% [14.05%] Black in the improver group vs 11.57% [12.72%] Black in the achiever group) and populations they treated (mean [SD] 74.57% [23.42%] White in the improver group vs 79.15% [22.18%] in the achiever group). Improvers vs achievers had moderately lower star ratings (mean [SD] overall star rating, 3.15 [1.30] vs 3.35 [1.32]), a higher proportion of for-profit ownership (1443 SNFs [78.9%] vs 9109 SNFs [70.7%]), more beds (mean [SD], 118.59 [59.3] vs 108.06 [59.0]), and residents with more deficiencies in activities of daily living (mean [SD] activities of daily living scores, 17.17 [1.99] vs 16.82 [2.10]) (Figure 1; eTable 2 in the Supplement). Small differences were found between the achiever and improvement groups in area deprivation (mean [SD] Area Deprivation Index score, 5.66 [1.63] vs 5.72 [1.67]), case mix index (mean [SD], 1.18 [0.13] vs 1.18 [0.17]), mean (SD) proportion of Medicaid bed-days (59.97% [22.80%] vs 59.53% [23.19%]), and operating margin (mean [SD], −0.38 [12.97] vs −0.76 [13.90]).

**Figure 2.**
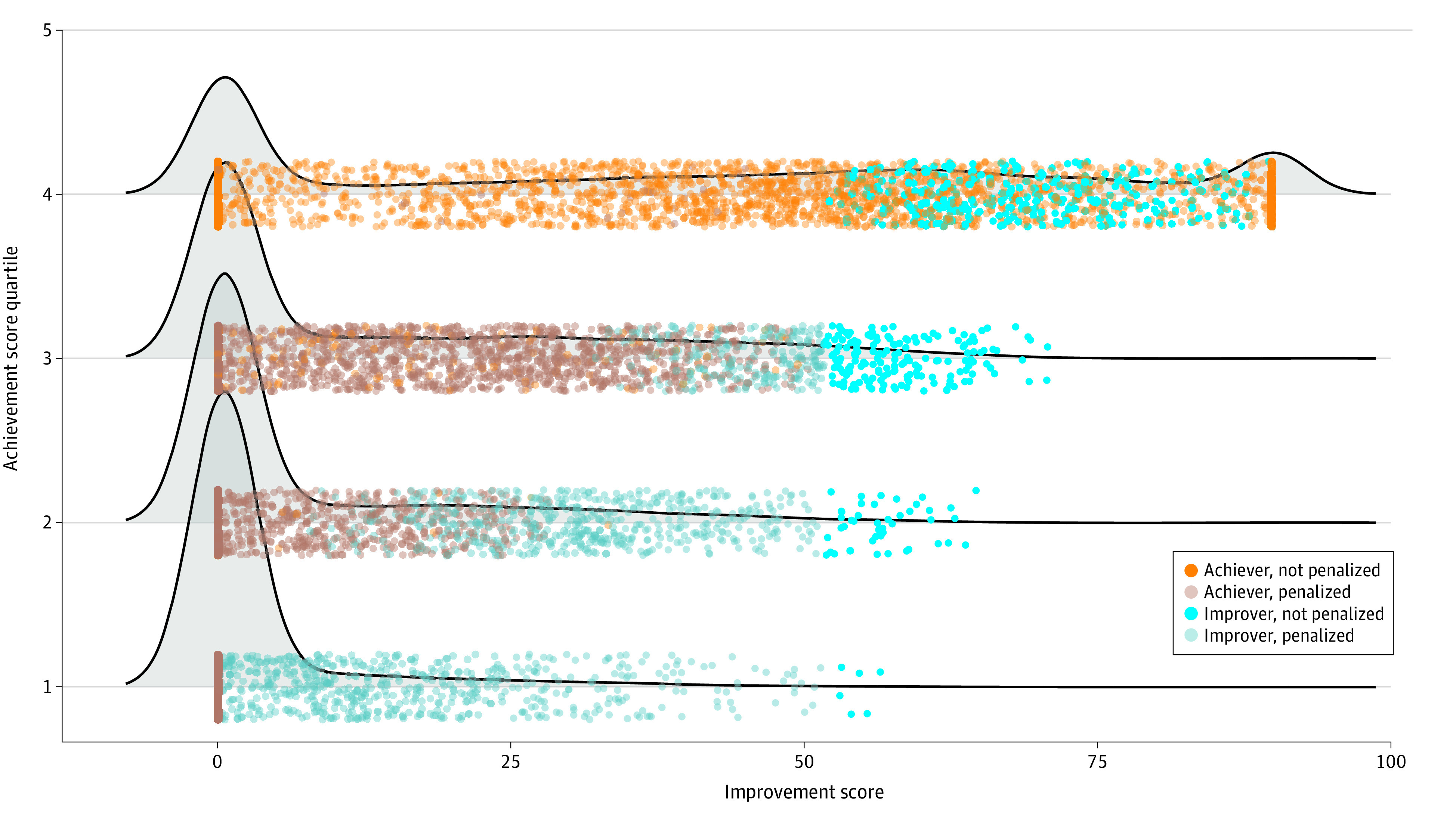
Distribution of Achievement and Improvement Scores in 2019 by Penalty Status Distribution of improvement scores (measured in 2017) by quartile of achievement scores (measured in 2015). *Achiever* refers to a skilled nursing facility (SNF) that had a higher achievement score than improvement score, and *improver* refers to an SNF that had a higher improvement score than achievement score. Penalty status was dichotomized such that SNFs were categorized regardless of the severity of the penalty or incentive. Table 1 lists the number of SNFs in the figure in each category by achievement score quartile.

Most improvers that were able to avoid financial penalties were already in the top half of SNFs at baseline (as measured by achievement score) (Figure 2 and Table 1). Only 6 facilities that started in the lowest quartile of achievement scores were able to improve enough to avoid being penalized, and only 52 facilities (0.7%) that started below the median achievement score avoided penalties. Differences in characteristics between improvers and achievers by quartile of achievement score are shown in the eTable 3 in the Supplement.

**Table 1.** Number of Skilled Nursing Facilities by Achievement and Improvement Scores in 2019 and Penalty Status^a^

Group	Achievement score quartile (low to high)				Total (n = 15 306)
	1	2	3	4	
**Achiever**					
Not penalized	0	32	310	3463	3805
Penalized	3256	3214	3126	56	9652
**Improver**					
Not penalized	6	46	167	306	525
Penalized	565	535	223	1	1324

^a^ Data are the number of skilled nursing facilities using improvement scores (measured in 2017) by quartile of achievement scores (measured in 2015). Achiever refers to a skilled nursing facility that had a higher achievement score than improvement score, and improver refers to a skilled nursing facility that had a higher improvement score than achievement score. Penalty status was dichotomized such that skilled nursing facilities were categorized regardless of the severity of the penalty or incentive.

The most improved SNFs in our sample (those in the top quartile of improvement scores) exhibited high achievement and improvement scores, resulting in a mean incentive multiplier of 1 (no bonus or penalty). In contrast, the SNFs that improved the most but started in the lower half of SNFs in terms of achievement score had a mean incentive multiplier of 0.98 (reflecting the maximum 2% penalty) (Table 2). In other words, the most improved SNFs that were in the bottom half of performers at baseline received a mean 2% financial penalty. When compared with the overall sample, the most improved SNFs were more likely to be in urban areas (2767 [74.9%] vs 10 901 [72.6%]) and had more beds (mean [SD] beds, 114.35 [65.91] vs 108.55 [60.13] beds), lower length of stay (mean [SD] days, 34.57 [21.96] vs 36.71 [24.57] days), and higher bed turnover (mean [SD] admissions per bed, 2.47 [2.35] vs 2.31 [2.28]). In contrast, compared with the overall sample, top improvers with below median baseline scores were less likely to be hospital based (36 [2.3%] vs 484 [3.4%]) and more likely to be for profit (1437 [79.7%] vs 10 691 [71.2%]) but similarly had a lower mean (SD) length of stay (34.97 [22.60] vs 36.71 [24.57] days) and higher bed turnover (mean [SD], 2.50 [2.41] vs 2.31 [2.28] number of admissions per bed).

**Table 2.** Comparison of SNFs in Top Quartile of Improvement Overall, SNFs in Top Quartile of Improvement With a Baseline Below Median Level of Performance, and Overall Sample^a^

Characteristic	Most improved SNFs (n = 3743)	Most improved with below median achievement (n = 1839)	Overall sample (N = 15 306)
SNF VBP program performance			
Baseline readmission rate, mean (SD), %	0.20 (0.02)	0.22 (0.02)	0.19 (0.02)
Performance readmission rate, mean (SD), %	0.18 (0.01)	0.20 (0.01)	0.19 (0.02)
Score, mean (SD)^b^			
Achievement (range, 1-100	56.40 (26.16)	13.15 (11.15)	32.65 (27.68)
Improvement (range, 1-100)	52.33 (20.39)	20.90 (14.37)	14.79 (24.43)
Performance (range, 1-100)	61.44 (20.77)	23.22 (13.56)	34.49 (27.48)
Incentive payment multiplier (range, 0.98-1.02)^b^	1.00 (0.01)	0.98 (0.01)	0.99 (0.01)
Facility characteristics, No. (%)			
Multifacility	2116 (60.1)	914 (58.0)	8490 (60.0)
Hospital based	115 (3.3)	36 (2.3)	484 (3.4)
For profit	2660 (72.0)	1437 (79.7)	10 691 (71.2)
Government owned	210 (5.7)	75 (4.2)	851 (5.7)
No. of changes in ownership, mean (SD)	1.72 (2.10)	1.66 (2.06)	1.67 (2.06)
No. of beds, mean (SD)	114.35 (65.91)	115.68 (55.83)	108.55 (60.13)
No. of admissions per bed, mean (SD)	2.47 (2.35)	2.50 (2.41)	2.31 (2.28)
Occupancy, mean (SD), %	80.52 (14.57)	80.44 (14.30)	79.93 (14.81)
Urban location, No. (%)	2767 (74.9)	1425 (79.1)	10 901 (72.6)
Net operating margin, mean (SD), %	−0.43 (13.32)	−1.23 (12.94)	−0.74 (13.87)
Star rating, mean (SD), (range, 1-5)^b^			
Overall	3.36 (1.32)	3.14 (1.31)	3.34 (1.32)
Quality	4.05 (1.07)	3.81 (1.16)	3.99 (1.10)
Survey	2.81 (1.26)	2.71 (1.25)	2.81 (1.26)
Staffing	3.08 (1.04)	2.97 (1.03)	3.10 (1.06)
Nurse staffing rating, mean (SD) (range, 1-5)^b^	3.26 (1.12)	3.08 (1.13)	3.28 (1.15)
Patient-level characteristics in facility			
Age, mean (SD), y	79.29 (6.03)	78.30 (6.19)	79.21 (6.35)
White (facility level), mean (SD), %	78.10 (22.57)	74.57 (23.50)	78.67 (22.38)
ADL Score, mean (SD)	16.96 (2.03)	17.12 (2.15)	16.83 (2.14)
Medicaid, mean (SD), %	59.16 (23.25)	61.58 (22.47)	59.71 (23.18)
Case-mix index, mean (SD)	1.19 (0.14)	1.18 (0.16)	1.18 (0.17)
Length of stay, mean (SD), d	34.57 (21.96)	34.97 (22.60)	36.71 (24.57)
County-level characteristics			
Black, mean (SD), %	12.66 (13.66)	14.74 (14.02)	11.96 (12.99)
Hispanic, mean (SD), %	13.99 (14.80)	14.59 (15.21)	13.80 (15.22)
White, mean (SD), %	79.14 (15.36)	77.16 (15.21)	80.04 (14.79)
ADI score, mean (SD)	5.65 (1.66)	5.62 (1.63)	5.72 (1.67)
Herfindahl-Hirschman Index score, mean (SD)	0.20 (0.24)	0.18 (0.24)	0.21 (0.24)
MA enrollment, mean (SD), %	31.0 (14.0)	32.0 (13.0)	31.0 (14.0)

Abbreviations: ADI, Area Deprivation Index; ADL, activities of daily living; MA, Medicare Advantage; SNF, Skilled Nursing Facility; SNF VBP, Skilled Nursing Facility Value-Based Purchasing.

^a^ Most improved SNFs are those with top quartile improvement scores. Most improved with below median achievement are those with achievement scores below 50.

^b^ All ranges were set by the Centers for Medicare & Medicaid Services to indicate low (smallest number) and high (largest number) scores.

**Figure 3.**
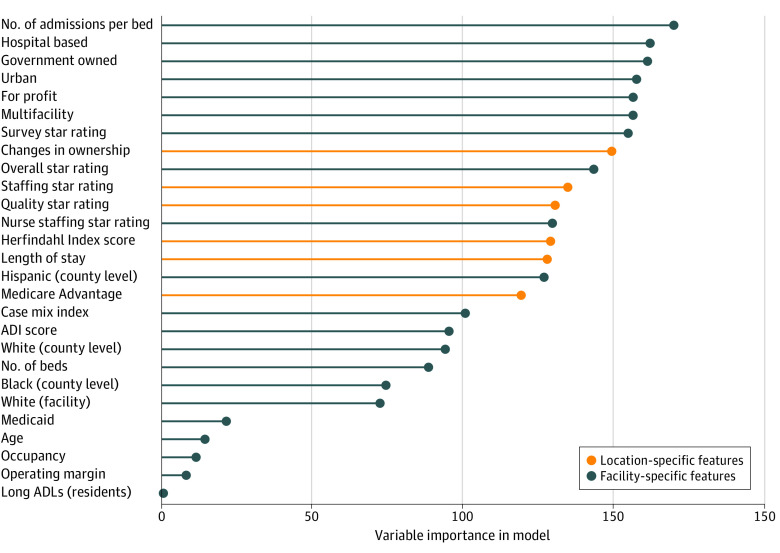
Relative Importance of Facility and County Characteristics in Predicting Whether a Skilled Nursing Facility (SNF) is an Improver vs an Achiever This variable importance plot was derived from a random forest model and excludes SNF value-based purchasing variables. Characteristics in orange are location-specific features, and characteristics in blue are facility-specific traits. The variable importance listed on the x-axis is the mean change in the importance measure and should be interpreted as demonstrating relative weights but is not meaningful in absolute terms. ADI indicates Area Deprivation Index; ADL, activities of daily living.

On the basis of the random forest plots, the most important predictors of improvement were SNF financial characteristics, such as the operating margin, occupancy rates, and how many stays were paid by Medicaid (Figure 3). Number of admissions per bed, as well as functional status, age, and race of SNF residents, were also important. These factors outweighed county-level characteristics, which in turn were more important than star ratings. Ownership characteristics were least important.

## Discussion

In this national cross-sectional study of all SNFs that accept Medicare payments for postacute care, 12.0% of facilities exhibited higher improvement scores than achievement scores, and 1 in 5 of those SNFs earned a financial bonus in the first year of financial incentives. In addition, 0.7% of SNFs that had high readmission rates at baseline improved enough to avoid financial penalties. Instead, top improvers with low baseline performance received the largest mean penalty in our sample (2.0%). These results suggest the program as currently structured rewards SNFs that are already high performing and penalizes those with low baseline performance, even if these SNFs achieved the top levels of improvement. In other words, as currently structured, the SNF VBP program helps the “rich get richer” (SNFs that are already high performing are rewarded) and financially punishes nearly all SNFs that were already poor (in terms of baseline performance), no matter what their level of improvement.

These findings contrast somewhat with evaluations of the SNF VBP program as a whole, which largely apply to achievers. For example, others^1,9^ have found that SNFs that avoided penalties under the SNF VBP program overall were larger, less likely to be for profit, and more likely to be rural. In addition, SNFs serving more minority and low-income populations have fared worse under the program overall. However, our findings are consistent with hospital value-based purchasing programs that shared a similar design (the Hospital VBP program and Hospital Readmissions Reduction Program) that incentivized both performance and improvement.^16^

These findings may be particularly important because there were signs that improvers could represent an important cohort to support in the SNF VBP program. Given evidence of inequities in allocation of high-value nursing home care among racial and ethnic minority groups,^6^ the higher proportion of Black populations in improvers and the counties where they reside represent important potential levers for improving outcomes in these populations.^17^ Similarly, larger, urban, for-profit SNFs that are part of a chain are those that have received significant negative press attention in the COVID-19 pandemic and have previous evidence of being of lower quality.^18,19^ Driving improvements in these facilities specifically could be a unique part of the improvement pathway in the SNF VBP program. Our finding that SNF financial characteristics (such as operating margin, occupancy rates, and Medicaid payment) were among the strongest predictors of improvement highlights the importance of aligning financial incentives with desired care improvements. It also highlights the potential sensitivity of SNFs to the financial impacts of value-based purchasing, suggesting this may be a strong lever to drive performance if deployed effectively.

The Medicare Payment Advisory Commission voted to approve recommendations for changes to the SNF VBP program during their April 2021 meeting, including broadening the mix of quality measures, making the program cost neutral to allow for more bonus payments, accounting for social risk of beneficiaries using a peer grouping approach, and recognizing the importance of developing patient experience metrics in SNFs.^20^ Whether these changes will be adopted by CMS is unclear. These changes would broaden how quality is measured beyond readmission rates, improve the all-cause readmission measure, and attempt to make comparisons across facilities more fair, particularly when it comes to measurement of social risk. These changes merit evaluation if adopted but are unlikely to address the major concern brought about by our findings: if the intent of the SNF VBP program is to drive improvement in performance through financial rewards, it is falling far short of its goals.

There are clear tradeoffs between incentivizing realistic improvement, rewarding high performers, and saving money for Medicare and other payers. The fact that only 60% of the 2% withheld from SNFs is redistributed (to ensure the program saves costs) and most of these funds went to achievers at baseline suggests improvement is not the top priority of the program. Policy changes that financially incentivize (rather than penalize) improvers who are low achievers at baseline may be more effective in driving continued improvement and addressing health inequities in the future, but doing so while also rewarding high-performing facilities under budget constraints may be difficult. At a minimum, improvers below the median achievement score could have their bonuses increased or penalties mitigated to encourage improvement. Other value-based purchasing programs that include an improvement pathway (such as the Hospital Value-Based Purchasing Program) were constrained to facilities that were already above the median in terms of baseline performance; thus, the effectiveness of such an approach is unknown.^16^ Given the impact of the SNF VBP program on SNF finances, analyses identifying potential unintended consequences of the SNF VBP program on SNF financial performance (and related concerns, such as staffing and closures, which may affect patient access) are needed. Furthermore, because many potential predictors of improvement are not observable in our data set, complementary approaches to identify how SNFs—particularly those with low performance at baseline—were able to improve are needed.^21,22,23^

The SNF VBP program is the first national value-based purchasing program in postacute care, but demonstration projects are also under way in other postacute settings, such as home health.^24^ Comparing and contrasting findings in these programs may reveal important insights into value-based purchasing design in postacute care. Thus, several features of the current SNF VBP program are important to note. First, the statute that created the program required it to be cost saving to the CMS, meaning that more SNFs are penalized than given a bonus by design. Second, the SNF VBP program is based on a single measure of all-cause, unplanned hospital readmissions, which contrasts with other VBP models that evaluate multiple metrics of performance and does not account for other outcomes for patients, such as transfers to other facilities or hospice. Finally, the SNF VBP program is based solely on Medicare fee-for-service claims. As Medicare Advantage becomes increasingly prevalent among Medicare enrollees, it is less clear how well the SNF VBP readmission measure will be able to reflect the true quality of the facility.

### Limitations

The most significant limitation of the current analysis is that it does not allow causal inference into the effect of the SNF VBP program on individual SNFs. In other words, although the analysis describes the as-treated population, the SNF VBP program may not be the only reason SNFs ended up in the achievement or improvement groups or why they scored a certain way on either scale, and other temporal trends could influence results. As an observational study, this work is also limited to evaluating SNF and market characteristics that are measured in existing data. Other unmeasured SNF characteristics, such as culture, leadership, relationships with hospitals, and quality improvement infrastructure, are not captured. Furthermore, the all-cause readmission measure used in SNFs does not account for the proportion of patients who are receiving hospice care or who have advance directives that might include wishes to not be hospitalized.

## Conclusions

Results of this cross-sectional study analyzing the start of the SNF VBP program indicate that only a nominal number of low-performing facilities were able to improve enough to avoid a financial penalty. When combined with previous results that indicate penalized SNFs overall are more likely to serve vulnerable populations, this outcome is a serious concern for the future of the program. However, improver SNFs may represent an important subset of SNFs to reward in value-based payment programs and may be particularly sensitive to changes in their financial bottom line. Iterative changes to the SNF VBP policy may help drive more intended consequences, in particular, driving improvement of low-performing facilities.

## Notes

Supplement. **eAppendix.** Brief Description of SNF VBP Program **eTable 1.** Data Sources Used in the Analysis **eTable 2.** Characteristics of SNF Achiever and Improver Groups **eTable 3.** Characteristics of SNF Achiever and Improver Groups by Quartile of Baseline Score
