# Supplementary material for: Skilled Nursing Facility Performance and Readmission Rates Under Value-Based Purchasing
Source: JAMA Netw Open. 2022 Feb 28;5(2):e220721. doi: 10.1001/jamanetworkopen.2022.0721 (PMC8886527; doi:10.1001/jamanetworkopen.2022.0721)
Supplement: Supplement. — eAppendix. Brief Description of SNF VBP Program eTable 1. Data Sources Used in the Analysis eTable 2. Characteristics of SNF Achiever and Improver Groups eTable 3. Characteristics of SNF Achiever and Improver Groups by Quartile of Baseline Score [file jamanetwopen-e220721-s001.pdf]

# Supplementary Online Content

Burke RE, Xu Y, Rose L. Skilled nursing facility performance and readmission rates under value-based purchasing. *JAMA Netw Open*. 2022;5(2):e220721. doi:10.1001/jamanetworkopen.2022.0721

**eAppendix.** Brief Description of SNF VBP Program
**eTable 1.** Data Sources Used in the Analysis
**eTable 2.** Characteristics of SNF Achiever and Improver Groups
**eTable 3.** Characteristics of SNF Achiever and Improver Groups by Quartile of Baseline Score

**This supplementary material has been provided by the authors to give readers additional information about their work.**

© 2022 Burke RE et al. *JAMA Network Open.*

**eAppendix.** Brief Description of SNF VBP Program

The SNF VBP Program was created under Protecting Access to Medicare Act of 2014 to improve the quality of care in skilled nursing facilities. Like other CMS value-base purchasing programs, CMS withholds a portion of Part A fee-for-service payments; for SNF VBP this is 2 percent. The program then awards incentive payments by redistributing 60 percent of these withheld funds, with the remaining 40 percent kept by CMS.

Facilities are assessed on an all-cause, all-condition risk-adjusted readmission measure. SNFs are scored on achievement score and an improvement score. The achievement score represents a SNF’s readmission rate in the baseline year relative to other facilities (FY2015 for the first year of the program). The improvement score represents the within-facility improvement two years after the achievement score year (FY2017 for the first year of the program). The SNF is then given the higher of the two scores for incentive payments. The lowest 40 percent of SNFs earn less than the 2 percent withheld by CMS. The remaining SNFs can earn the exact amount withheld (also given to facilities with very low numbers of patients and readmissions) or more than the amount withheld.

All SNFs that are reimbursed under the CMS SNF prospective payment system are subject to the program. The first award incentives affected FY19 payments.

© 2022 Burke RE et al. *JAMA Network Open.*

**eTable 1.** Data Sources Used in the Analysis

| Data Source | Description | Variables used in the analysis |
|---|---|---|
| Skilled nursing facility value-based purchasing program performance data | Data regarding all-cause readmission rates and performance under the SNF VBP program at a facility level for the baseline and performance periods | All-cause, risk-standardized readmission rates, improvement scores, achievement scores, performance scores, incentive multipliers |
| CMS Provider of Services file and LTCFocus | Provides data regarding skilled nursing facility characteristics (CMS provider of services) and patient characteristics (LTCFocus) at a facility level | Facility characteristics listed in Table 1 (CMS Provider of Services), Patient characteristics listed in Table 1 (LTCFocus) other than star ratings and net operating margin. In addition, the location of nursing homes in the Provider of Services file was used to calculate a Hefindahl-Hirschman index. |
| Nursing Home Compare | Data regarding SNF quality as reflected in aggregate star ratings | Overall star rating, as well as component parts (quality star rating, survey star rating, staffing star rating, and registered nursing staffing in particular) |
| SNF cost reports | Data regarding SNF revenues, operating costs, and operating margin | Net operating margin |
| 2015 US Census residential population estimates | Data regarding county-level characteristics | Proportion of Medicare beneficiaries of different races/ethnicities. These data also are used to calculate the Area Deprivation Index which was aggregated to the county level. |
| Medicare Advantage enrollment | Medicare Advantage enrollment data at the county level | Proportion of Medicare Advantage enrollees at the county level |
| Post-acute care and hospice provider utilization and payment public use files | Data regarding total Medicare payments to SNF | Total Medicare payments were used to estimate the size of the financial penalty for each SNF |

© 2022 Burke RE et al. *JAMA Network Open.*

**eTable 2.** Characteristics of SNF Achiever and Improver Groups

| **Characteristic** | Achiever group mean/% N=13,110 | Improver group mean/% N=1,839 | Standardized difference |
|---|---|---|---|
| **SNF VBP performance** | | | |
| Baseline readmission rate | 0.19 (0.01) | 0.22 (0.01) | -2.499 |
| Performance readmission rate | 0.19 (0.02) | 0.20 (0.01) | -0.191 |
| Achievement Score | 33.57 (28.40) | 25.00 (23.29) | 0.308 |
| Improvement Score | 11.64 (23.23) | 37.77 (21.46) | -1.135 |
| Performance Score | 33.57 (28.40) | 37.77 (21.46) | -0.152 |
| Incentive Payment Multiplier | 0.99 (0.01) | 0.99 (0.01) | -0.086 |
| **Facility characteristics** | | | |
| Multi-facility | 7,376 (60.87%) | 1,007 (57.74%) | 0.064 |
| Hospital-based | 397 (3.28%) | 39 (2.24%) | 0.060 |
| For-profit | 9,109 (70.69%) | 1,443 (78.94%) | -0.183 |
| Government-owned | 724 (5.62%) | 74 (4.05%) | 0.069 |
| # Changes in ownership | 1.67 (2.06) | 1.82 (2.08) | -0.073 |
| # of Beds | 108.06 (59.00) | 118.59 (59.33) | -0.178 |
| # of Admissions/Bed | 2.30 (2.27) | 2.61 (2.44) | -0.135 |
| % Occupancy | 79.80 (14.84) | 80.50 (14.31) | -0.047 |
| Urban location | 9,334 (72.44%) | 1,411 (77.19%) | -0.107 |
| Net operating margin | -0.76 (13.90) | -0.38 (12.97) | -0.028 |
| Overall star rating | 3.35 (1.32) | 3.15 (1.30) | 0.154 |
| Quality star rating | 4.01 (1.09) | 3.85 (1.15) | 0.151 |
| Survey star rating | 2.82 (1.26) | 2.69 (1.24) | 0.097 |
| Staffing star rating | 3.10 (1.06) | 2.96 (0.99) | 0.134 |
| RN Staffing Rating | 3.30 (1.15) | 3.08 (1.09) | 0.187 |
| **Patient characteristics in facility** | | | |
| Age, mean (years) | 79.29 (6.03) | 78.30 (6.19) | 78.48 (10.41) |
| % White, facility-level | 79.15 (22.18) | 74.57 (23.42) | 0.205 |
| Long ADL | 16.82 (2.10) | 17.17 (1.99) | -0.168 |
| % Medicaid | 59.53 (23.19) | 59.97 (22.80) | -0.019 |
| Case-mix index | 1.18 (0.17) | 1.18 (0.13) | -0.033 |
| Median length of stay, days | 36.33 (23.96) | 33.30 (20.16) | 0.129 |
| **County characteristics** | | | |
| % White, county-level | 80.43 (14.59) | 76.68 (15.54) | 0.255 |
| % Black, county-level | 11.57 (12.72) | 15.48 (14.50) | -0.302 |
| % Hispanic, county-level | 13.75 (15.25) | 14.56 (15.17) | -0.053 |
| ADI score | 5.72 (1.67) | 5.66 (1.63) | 0.037 |
| Hirschman-Herfindahl Index | 0.21 (0.24) | 0.19 (0.24) | 0.060 |
| % MA enrollment (county) | 0.31 (0.14) | 0.32 (0.13) | -0.071 |

Source: Author analysis of CMS data. Notes: Means (for binary variables) and proportions (for categorical variables) of key SNF characteristics for the achiever and improver groups.

© 2022 Burke RE et al. *JAMA Network Open.*

**eTable 3.** Characteristics of SNF Achiever and Improver Groups by Quartile of Baseline Score

| **Characteristic** | Quartile 1 | | | Quartile 2 | | | Quartile 3 | | | Quartile 4 | | |
|---|---|---|---|---|---|---|---|---|---|---|---|---|
| | A N=3256 | I N=573 | p-value | A N=3215 | I N=508 | p-value | A N=3130 | I N=390 | p-value | A N=3509 | I N=306 | p-value |
| **SNF VBP performance** | | | | | | | | | | | | |
| Baseline readmission rate | 0.19 (0.02) | 0.23 (0.01) | -2.23 | 0.19 (0.01) | 0.22 (0.01) | -2.89 | 0.18 (0.01) | 0.22 (0.01) | -2.81 | 0.18 (0.02) | 0.22 (0.01) | -2.56 |
| Performance readmission rate | 0.22 (0.01) | 0.21 (0.01) | 0.38 | 0.20 (0.00) | 0.20 (0.00) | -0.13 | 0.19 (0.00) | 0.19 (0.00) | 0.04 | 0.17 (0.01) | 0.18 (0.00) | -0.50 |
| Achievement Score | 0.18 (1.00) | 0.33 (1.33) | -0.14 | 19.24 (7.08) | 18.32 (7.08) | 0.13 | 40.62 (5.95) | 40.84 (5.85) | -0.04 | 71.37 (15.46) | 63.67 (9.82) | 0.51 |
| Improvement Score | 0.00 (0.00) | 17.12 (12.74) | -3.44 | 1.84 (5.12) | 33.02 (12.02) | -4.67 | 8.36 (13.05) | 50.61 (8.34) | -3.35 | 34.12 (33.19) | 69.06 (9.27) | -1.10 |
| Performance Score | 0.18 (1.00) | 17.12 (12.74) | -3.38 | 19.24 (7.08) | 33.02 (12.02) | -1.72 | 40.62 (5.95) | 50.61 (8.34) | -1.60 | 71.37 (15.46) | 69.06 (9.27) | 0.15 |
| Incentive Payment Multiplier | 0.98 (0.00) | 0.98 (0.00) | -1.53 | 0.98 (0.00) | 0.99 (0.01) | -1.99 | 0.99 (0.00) | 1.00 (0.01) | -1.69 | 1.01 (0.01) | 1.01 (0.00) | -0.12 |
| **Facility characteristics** | | | | | | | | | | | | |
| Multi-facility | 1,873 (62.23%) | 281 (52.33%) | 0.20 | 1,811 (61.02%) | 321 (59.33%) | 0.03 | 1,717 (60.12%) | 231 (62.10%) | -0.04 | 1,975 (60.16%) | 174 (59.18%) | 0.02 |
| Hospital-based | 73 (2.43%) | 12 (2.23%) | 0.01 | 77 (2.59%) | 10 (1.85%) | 0.05 | 110 (3.85%) | 9 (2.42%) | 0.08 | 137 (4.17%) | 8 (2.72%) | 0.07 |
| For-profit | 2,459 (76.77%) | 463 (81.23%) | -0.11 | 2,249 (71.46%) | 463 (81.37%) | -0.22 | 2,125 (69.42%) | 286 (74.29%) | -0.11 | 2,276 (65.52%) | 231 (75.99%) | -0.22 |
| Government-owned | 109 (3.40%) | 20 (3.51%) | <-0.01 | 182 (5.78%) | 17 (2.99%) | 0.12 | 198 (6.47%) | 25 (6.49%) | <-0.01 | 235 (6.76%) | 12 (3.95%) | 0.11 |
| # Changes in ownership | 1.72 (2.04) | 1.76 (2.01) | -0.02 | 1.69 (2.08) | 1.84 (2.08) | -0.07 | 1.67 (2.10) | 1.95 (2.21) | -0.13 | 1.60 (2.04) | 1.74 (2.07) | -0.07 |
| # of Beds | 115.31 (53.49) | 120.02 (54.58) | -0.09 | 101.84 (56.20) | 117.62 (54.67) | -0.28 | 101.54 (56.80) | 119.52 (72.76) | -0.31 | 112.72 (66.52) | 116.58 (57.16) | -0.06 |
| # of Admissions/Bed | 2.64 (2.40) | 2.93 (2.67) | -0.12 | 1.99 (1.94) | 2.55 (2.55) | -0.27 | 1.98 (2.05) | 2.39 (2.11) | -0.20 | 2.55 (2.51) | 2.43 (2.12) | 0.05 |
| % Occupancy | 79.66 (14.62) | 81.92 (13.40) | -0.16 | 79.34 (15.13) | 79.80 (14.56) | -0.03 | 79.39 (15.02) | 79.73 (14.46) | -0.02 | 80.70 (14.57) | 80.18 (15.12) | 0.04 |
| Urban location | 2,472 (77.18%) | 453 (79.47%) | -0.06 | 2,217 (70.45%) | 457 (80.32%) | -0.22 | 2,117 (69.14%) | 283 (73.51%) | -0.10 | 2,528 (72.77%) | 218 (71.71%) | 0.02 |
| Net operating margin | -0.77 (13.53) | -0.87 (14.68) | 0.01 | -1.23 (12.38) | -0.64 (11.33) | -0.05 | -0.68 (16.60) | -0.58 (10.69) | -0.01 | -0.41 (12.89) | 1.32 (14.78) | -0.13 |
| Overall star rating | 3.18 (1.32) | 3.18 (1.28) | <-0.01 | 3.29 (1.31) | 3.04 (1.33) | 0.19 | 3.31 (1.33) | 3.15 (1.29) | 0.12 | 3.62 (1.28) | 3.30 (1.31) | 0.24 |
| Quality star rating | 3.81 (1.15) | 3.81 (1.17) | <0.01 | 3.96 (1.11) | 3.72 (1.16) | 0.22 | 3.99 (1.10) | 3.96 (1.13) | 0.03 | 4.26 (0.96) | 4.01 (1.08) | 0.26 |
| Survey star rating | 2.74 (1.25) | 2.72 (1.22) | 0.01 | 2.78 (1.26) | 2.64 (1.28) | 0.11 | 2.78 (1.27) | 2.62 (1.20) | 0.12 | 2.96 (1.26) | 2.83 (1.25) | 0.10 |
| Staffing star rating | 2.98 (1.04) | 3.02 (0.95) | -0.04 | 3.07 (1.05) | 2.92 (1.02) | 0.14 | 3.09 (1.07) | 2.97 (1.01) | 0.11 | 3.27 (1.04) | 2.93 (1.02) | 0.32 |

© 2022 Burke RE et al. *JAMA Network Open.*

| | | | | | | | | | | | | |
|---|---|---|---|---|---|---|---|---|---|---|---|---|
| RN Staffing Rating | 3.12 (1.14) | 3.05 (1.10) | 0.06 | 3.26 (1.15) | 3.08 (1.08) | 0.16 | 3.30 (1.16) | 3.12 (1.09) | 0.16 | 3.49 (1.10) | 3.10 (1.13) | 0.35 |
| **Patient characteristics in facility** | | | | | | | | | | | | |
| Age, mean (years) | 78.86 (5.90) | 79.01 (5.12) | -0.03 | 78.81 (6.90) | 78.33 (5.55) | 0.07 | 79.20 (6.50) | 78.69 (5.66) | 0.08 | 80.19 (5.94) | 79.22 (5.68) | 0.16 |
| % White, facility-level | 75.15 (23.61) | 74.43 (22.89) | 0.03 | 79.75 (21.70) | 73.18 (24.47) | 0.30 | 80.14 (21.62) | 75.33 (22.37) | 0.22 | 81.41 (21.23) | 76.39 (23.62) | 0.23 |
| Long ADL | 17.14 (1.97) | 17.33 (1.94) | -0.10 | 16.67 (2.32) | 17.10 (2.01) | -0.19 | 16.63 (2.17) | 17.09 (2.01) | -0.21 | 16.82 (1.92) | 17.14 (1.99) | -0.16 |
| % Medicaid | 59.17 (22.93) | 58.19 (22.45) | 0.04 | 61.74 (22.82) | 61.40 (23.40) | 0.02 | 61.15 (22.51) | 60.96 (22.22) | 0.01 | 56.44 (23.97) | 59.34 (22.92) | -0.12 |
| Case-mix index | 1.19 (0.15) | 1.19 (0.13) | 0.02 | 1.17 (0.18) | 1.18 (0.13) | -0.08 | 1.17 (0.18) | 1.18 (0.13) | -0.08 | 1.19 (0.15) | 1.18 (0.12) | 0.05 |
| Median length of stay, days | 33.02 (20.28) | 31.69 (18.03) | 0.07 | 39.54 (27.09) | 34.36 (21.71) | 0.20 | 39.22 (26.14) | 34.43 (21.46) | 0.19 | 33.97 (21.27) | 32.86 (19.04) | 0.05 |
| **County characteristics** | | | | | | | | | | | | |
| % White, county-level | 77.94 (14.48) | 74.75 (15.57) | 0.22 | 80.96 (14.36) | 77.38 (15.24) | 0.25 | 81.53 (14.68) | 77.35 (15.81) | 0.28 | 81.30 (14.56) | 78.14 (15.47) | 0.22 |
| % Black, county-level | 13.96 (13.25) | 17.22 (14.81) | -0.24 | 11.34 (12.61) | 14.68 (13.82) | -0.26 | 10.84 (12.64) | 15.05 (14.86) | -0.33 | 10.21 (12.09) | 14.29 (14.45) | -0.33 |
| % Hispanic, county-level | 15.34 (16.49) | 15.17 (15.21) | 0.01 | 13.01 (14.96) | 13.99 (15.02) | -0.07 | 13.19 (15.23) | 14.91 (16.11) | -0.11 | 13.44 (14.19) | 14.08 (14.11) | -0.05 |
| ADI score | 5.70 (1.66) | 5.55 (1.56) | 0.09 | 5.78 (1.66) | 5.65 (1.64) | 0.08 | 5.77 (1.66) | 5.69 (1.60) | 0.04 | 5.64 (1.70) | 5.84 (1.78) | -0.11 |
| Hirschman-Herfindahl Index | 0.18 (0.22) | 0.18 (0.24) | 0.02 | 0.22 (0.25) | 0.18 (0.23) | 0.16 | 0.23 (0.25) | 0.21 (0.26) | 0.07 | 0.20 (0.23) | 0.22 (0.25) | -0.08 |
| % MA enrollment (county) | 0.31 (0.13) | 0.33 (0.13) | -0.10 | 0.31 (0.14) | 0.32 (0.13) | -0.06 | 0.31 (0.14) | 0.31 (0.14) | -0.04 | 0.30 (0.14) | 0.31 (0.13) | -0.04 |

Source: Author analysis of CMS data. Notes: Means (for binary variables) and proportions (for categorical variables) of key SNF characteristics for the achiever (“A”) and improver (“I”) groups within each quartile of achievement (baseline) score. The final columns give the standardized mean difference between the achiever and improver groups. Facilities without a baseline score are dropped.

© 2022 Burke RE et al. *JAMA Network Open.*
